# Isolation and transcriptome analysis of a biotechnologically promising Black Sea protist, *Thraustochytrium aureum ssp. strugatskii*

**DOI:** 10.7717/peerj.12737

**Published:** 2022-03-09

**Authors:** Dmitrii K. Konstantinov, Aleksei Menzorov, Olga Krivenko, Alexey V. Doroshkov

**Affiliations:** 1 Novosibirsk State University, Novosibirsk, Russia; 2 Institute of Cytology and Genetics Siberian Branch of the Russian Academy of Sciences, Novosibirsk, Russia; 3 A.O. Kovalevsky Institute of Biology of the Southern Seas of RAS, Sevastopol, Russia; 4 Siberian Federal University, Krasnoyarsk, Russia

**Keywords:** *Thraustochytrium*, Fatty acids, RNA-seq, Transcriptome assembly, Marine protists

## Abstract

**Background:**

Marine protists are an important part of the ocean ecosystem. They may possess unique sets of biosynthetic pathways and, thus, be promising model organisms for metabolic engineering for producing substances for the pharmaceutical, cosmetic, and perfume industries. Currently, full-genome data are available just for a limited number of protists hampering their use in biotechnology.

**Methods:**

We characterized the morphology of a new cultured strain of Thraustochytriaceae isolated from the Black Sea ctenophore *Beroe ovata* using phase-contrast microscopy. Cell culture was performed in the FAND culture medium based on fetal bovine serum and DMEM. Phylogenetic analysis was performed using the 18S rRNA sequence. We also conducted a transcriptome assembly and compared the data with the closest species.

**Results:**

The protist belongs to the genus *Thraustochytrium* based on the 18S rRNA sequence analysis. We designated the isolated protist as *T. aureum ssp. strugatskii*. The closest species with the genome assembly is *Schizochytrium aggregatum*. Transcriptome analysis revealed the majority of the fatty acid synthesis enzymes.

**Conclusion:**

Our findings suggest that the *T. aureum ssp. strugatskii* is a promising candidate for biotechnological use. Together with the previously available, our data would allow the establishment of an accurate phylogeny of the family Thraustochytriaceae. Also, it could be a reference point for studying the evolution of the enzyme families.

## Introduction

Protists are an important part of marine ecosystems (Massana et al., 2015). Analysis of a large set of protist species would allow us to update their systematic position, expand our knowledge of marine ecology (Strassert et al., 2019), and find promising model organisms for use in metabolic engineering (Singh et al., 2014).

Labyrinthulomycetes are heterotrophic protists widely spread in the various marine environments (Mystikou et al., 2014; Pan, Del Campo & Keeling, 2017) and also found in freshwater (Takahashi et al., 2014; Anderson & Cavalier-Smith, 2012). These fungus-like protists belong to Stramenopiles, produce heterokont biflagellate zoospores in the life cycle, and have unique cell organelle called bothrosome, which forms specific filaments of the ectoplasmic network and is considered an apomorphic trait of the group (Porter, 1969; Moss, 1985; Moro et al., 2003; Hamamoto & Honda, 2019). Such cytoplasmic threads provide effective osmotrophic absorption of nutrients, the main nutritional mode for Labyrinthulomycetes (Hamamoto & Honda, 2019). They live as epibionts or endobionts associated with plants or animals as parasites, mutualists, commensals, or saprobes using live or dead organic matter for nutrition (Raghukumar, 2002; Bennet et al., 2017). Their ecological role is usually associated with the decomposition of organic matter and nutrient recycling in the estuarine, coastal, and open ocean ecosystems, including deep-water and anoxic environments (Raghukumar, 2002; Raghukumar & Damare, 2011; Singh et al., 2014).

Taxonomy and phylogenetic relationships within Labyrinthulomycetes at the level of higher taxa are mostly resolved (Tsui et al., 2009; Doi & Honda, 2017). At the lower taxonomic level, it is constantly refined with the emergence of new molecular data (Bennet et al., 2017; Pan, Del Campo & Keeling, 2017). There are two main clades: (a) holocarpic thraustochytrids and (b) plasmodial labyrinthulids and aplanochytrids (Bennet et al., 2017). The most diverse extensively studied family Thraustochytriaceae contains 14 genera (Dellero et al., 2018). Morphological similarity between the Thraustochytrids family members causes nomenclatural errors (Andersen & Ganuza, 2018). In particular, 18S rRNA sequences of *Thraustochytrium* and *Ulkenia* form several phylogenetic clades. Probably there are several genetically diverse though morphologically similar species (Yokoyama & Honda, 2007). The use of both 18S rRNA sequences and whole-genome data should clarify their systematic position (Liang et al., 2019; Ju et al., 2019).

Thraustochytrids accumulate a lot of triacylglycerols (Abida et al., 2015; Dellero et al., 2018). They also accumulate significant amounts of polyunsaturated fatty acids (*ω*-3 LC-PUFAs) with very long chains, in particular omega-3-docosahexaenoic acid (DHA, 22:6 *ω*3) (Manikan et al., 2015; Aasen et al., 2016; Fan et al., 2007) and eicosapentaenoic acid (EPA, 20:5 *ω*3) (Chang et al., 2012). EPA and DHA play a key role in cardiovascular health (Lopez-Huertas, 2010) and DHA has a significant role in an infant’s brain and eye development (Mun et al., 2019). Also, these molecules participate in cholesterol and prostaglandin level regulation, thromboxane and leukotriene biosynthesis, and have a role in the development of such diseases as atherosclerosis, asthma, thrombosis, arthritis, and a wide range of tumors (Funk, 2001; Qiu, 2003; Damude & Kinney, 2007). Some Thraustochytrid strains produce arachidonic acid (AA, 20:4 *ω*6) (Yang et al., 2010; Ou et al., 2016), which is essential for many animals (Muskiet et al., 2004). Moreover, saturated and unsaturated fatty acids could be used as biofuel or have the potential for usage in biotechnology (Singh et al., 2014). The fishery is currently the major source of omega-3 polyunsaturated fatty acids (Chang et al., 2012). However, the state of the world fish stocks, ecological consequences of the industrial fishery, and fish oil contamination are troubling (Pauly et al., 2002). Thus, the study of this family biodiversity is important for increasing knowledge of the biochemical pathways of fatty acids in the environment.

Currently, 15 species of Labyrinthulomycetes are known in the Ponto-Caspian basin (Kopytina, 2018, Kopytina, 2019a; Kopytina, 2019b; Popova et al., 2019). Overall, this taxon’s diversity, distribution, and ecology in the Black Sea have not been studied yet. In this study, we characterized a new protist isolated from the Black Sea ctenophore *Beroe ovata*. Morphological, 18S rRNA, and transcriptome analyses allowed us to show that it belongs to the family Thraustochytriaceae, genus *Thraustochytrium*. We designated it as *T. aureum ssp. strugatskii*.

## Materials & Methods

### Cell culture and cryoconservation

The *T. aureum ssp. strugatskii* was isolated from dissociated *Beroe ovata*. Animals were collected in the Black Sea using plastic containers and maintained at room temperature (22–24 °C) in an aquarium for six days. *B. ovata* were dissociated using the modified protocol from Vandepas et al. (2017). Briefly, animals were cut with scissors in 17% artificial seawater (ASW: artificial sea salt, Red Sea Coral Pro, Red Sea International, Eilat, Israel, dissolved in distilled water) and centrifuged. An equal volume of 0.25% Trypsin-EDTA was added to the sediment, cells were dissociated using an orbital shaker at 500 rpm for 15 min at room temperature. 10% V/V FBS was added to inactivate trypsin. The resulting homogenate was centrifuged twice in ASW for 5 min at 300 g at room temperature to pellet cells. Cells were resuspended and cultured in FAND culture medium: 17‰ ASW, 5% FBS, 5% DMEM (prepared from powder on 17‰ ASW), x0.05 NEAA, x1 PenStrep, all reagents from Thermo Fisher Scientific, USA.

Cells were cultured in 6-well cell culture plates (Thermo Fisher Scientific, USA) with the mechanical passage: scraped from plastic by 1 ml pipet tip, pipetted hard several times, and an aliquot put into the next well with dilution ratio from 1:10 to 1:1000.

Cryoconservation was performed in a freezing medium containing 90% KnockOut Serum Replacement (Thermo Fisher Scientific, USA) and 10% DMSO (Sigma-Aldrich, USA) in Mr. Frosty Freezing Container (Thermo Fisher Scientific, USA) at −80 °C overnight; then cells were transferred to liquid nitrogen for long-term storage.

Cell culture was performed at the Collective Center of ICG SB RAS “Collection of Pluripotent Human and Mammalian Cell Cultures for Biological and Biomedical Research” (https://ckp.icgen.ru/cells/; http://www.biores.cytogen.ru/brc_cells/collections/ICG_SB_RAS_CELL). The cell line is available at the Collective Center.

### Analysis of morphology and growth rate

Morphology was observed daily using phase-contrast microscopy under an inverted microscope Observer Z1 (ZEISS, Germany) using a 40×lens and differential interference contrast (DIC). The growth rate was recorded using the automated cell culture and analysis system Cell-IQ (CM Technologies Oy, Finland) using 40×lens and phase contrast technology. Images were taken every 20 min. Cell count and cell population density level were estimated using ImageJ2 (Rueden et al., 2017).

### DNA isolation and rRNA sequencing

Genomic DNA was isolated from cells using a PCR buffer with nonionic detergents (PBND) by the protocol adapted from Perkin Elmer Cetus (Higuchi, 1989). We used previously published universal primer sequences for eukaryotic 18S rRNA: F-566 CAG CAG CCG CGG TAA TTC C and R-1200 CCC GTG TTG AGT CAA ATT AAG C (Hadziavdic et al., 2014). PCR was performed using BioMaster HS-Taq PCR-Color (2 ×) (Biolabmix, Russia) in 10 µL reaction volume. Amplification was carried out in a T100 thermal cycler (Bio-Rad, USA) using the following program: 95 °C for 5 min, 40 cycles consisting of 95 °C for 15 s, 60 °C for 15 s, 72 °C for 30 s, and a final extension step of 72 °C for 5 min. PCR products were precipitated by ethanol and amplified with the BigDye Terminator v3.1 Cycle Sequencing Kit (Thermo Fisher Scientific, USA) according to the manufacturer’s recommendations. Sequencing was done at the “Molecular and Cellular Biology” core facility of the IMCB SB RAS, Russia. Sequence analysis was made using Standard Nucleotide BLAST, blastn (https://blast.ncbi.nlm.nih.gov/), nucleotide collection (nr/nt) database.

### RNA isolation and sequencing

Total RNA was extracted from preliminarily centrifuged cells using a Direct-Zol RNA MiniPrep Kit according to the manufacturer’s recommendations (Zymo Research Corporation, Irvine, CA, USA). Three biological replicates were prepared. One of the libraries was prepared from a sample enriched with small motile cells, presumably zoospores. RNA was purified using RNAClean XP (Beckman Coulter, Brea, CA, USA) and dissolved in 50 µl of water. RNA quality was measured by the 2100 Bioanalyzer instrument using RNA 6000 Nano Kit (Agilent, Santa Clara, CA ,USA) according to the manufacturer’s recommendations.

Library preparation and barcoding were made with the Illumina TruSeq Stranded mRNA Kit (Illumina Inc, San Diego, CA, USA), single index primers were used for barcoding. Poly(A) RNA enrichment was performed according to the manufacturer’s recommendations, poly(A) fragmentation was four min to produce longer inserts. The first and second cDNA strand synthesis, 3′ adenylation, and adapter ligation were performed strictly according to the manufacturer’s recommendations. Ligation products were purified using AmPureXP (Beckman Coulter, Brea, CA). 10 cycles of library amplification were performed using SimpliAmp Thermal Cycler (Thermo Fisher Scientific, Waltham, MA, USA). Final library purification was achieved with AmPureXP using 0.9 V of magnetic beads, the libraries were washed by 50 µl of water. Quality, concentration, and molarity were measured by the 2100 Bioanalyzer instrument using the High Sensitivity DNA Kit (Agilent, Santa Clara, CA, USA). The libraries were diluted 10 times before the measurement. The DNA concentrations for three replicas were 5 (motile cell enrichment), 17, and 25 nM, respectively.

Library sequencing was performed with other samples on the NextSeq 550 Sequencing System with NextSeq 500 Mid Output v2 Kit (150 cycles) (Illumina Inc.). We used 150 bp forward single end reads. The number of quality-filtered reads was 19.7 mln, average quality was 30–31.

The raw sequence data sets are available in NCBI BioProject repository, accession number: PRJNA669615. 18S rRNA sequence is deposited in NCBI GenBank (accession number: MW165782).

### Transcriptome assembly

Quality control was performed using FastQC v0.11.5 (Andrews, 2010). Adapters and primers were removed in Trimmomatic v.0.3 (Bolger, Lohse & Usadel, 2014) using HEADCROP and ILLUMINACLIP parameters (with built-in SE-library of TruSeq3-SE index adapters). SLIDINGWINDOW:3:20 and MINLEN:36 parameters were used to remove short and low quality reads. Contig assembly was performed using Trinity (Grabherr et al., 2011) with default settings on merged libraries.

### Gene annotation

The TransDecoder (https://github.com/TransDecoder) was used for open reading frames search. Markov chains from the Pfam database (Pfam v32) (Finn et al., 2016) were used to find and describe protein domains. GO terms were assigned to Pfam domains according to a previously published protocol (http://current.geneontology.org/ontology/external2go/pfam2go).

Annotation of biochemical pathways was done using KEGG PATHWAY database (Kanehisa, 2002). *T. aureum ssp. strugatskii* proteins were blasted against the closest species from KEGG (*T. aureum* and *T. roseum*) and three animals (*Mus musculus*, *Drosophila melanogaster*, and *Caenorhabditis elegans*). The best hit, its KO (KEGG Orthology) group, and biochemical pathway affiliation were taken.

### Phylogenetic analysis of 18S gene

Multiple alignments of the 18S gene were performed using MAFFT v.7 (Katoh & Standley, 2013) with “–add”, “–auto”, and “–keeplength” parameters. Analysis of molecular evolution was carried out with the SAMEM v.0.82 pipeline (Gunbin et al., 2012). FastTree v.2.1.1 (Price, Dehal & Arkin, 2010) was used for estimating the primary topology. The construction of the final phylogenetic tree based on the previously generated substitution model was carried out by Phyml (Si Quang, Gascuel & Lartillot, 2008) by optimization of primary tree topology and branch lengths. We used the aLRT procedure to test the stability of the tree branching points. Tree visualization and topology analysis were performed in FigTree v.1.4.2 (Rambaut & Drummond, 2008, http://tree.bio.ed.ac.uk/software/figtree/), Archaeopteryx (https://sites.google.com/site/cmzmasek/home/software/archaeopteryx), and ETE toolkit (Huerta-Cepas, Serra & Bork, 2016).

To accurately determine the relationship of species, we used the combination of phylogenetic trees for individual proteins in the SuperTriplets program (Ranwez, Criscuolo & Douzery, 2010). To identify ortholog protein sets, we used blastp (e-value <0.0001, identity >0.6) for the proteoms of seven species (*T. aureum ssp. Strugatskii*, *S. aggregatum*, *Aurantiochytrium limacinum*, *Hondaea fermentalgiana*, *Aplanochytrium kerguelense*, *Aurecoccus anophagefferens*, and *Phytophthora infestans*). We selected proteins with a target one-to-one, meaning that all proteins in the set have found each other unequivocally in all pairs for all types in BLAST request. Methods of multiple alignments and phylogenetic analysis are described above.

## Results

### Morphology and life cycle

We isolated protists from dissociated *B. ovata*. Cell suspensions from a single animal were plated on 12- or 24-well plates. We observed motile *T. aureum ssp. strugatskii* cells in about half of the wells, thus the number of protists per animal was probably low. Interestingly, in a separate experiment, we were able to isolate and culture *T. kinnei*, *Aplanochytrium*, and *Cafeteria*. Species identity was based on rRNA sequence analysis (Supplemental Information 1).

**Figure 1:**
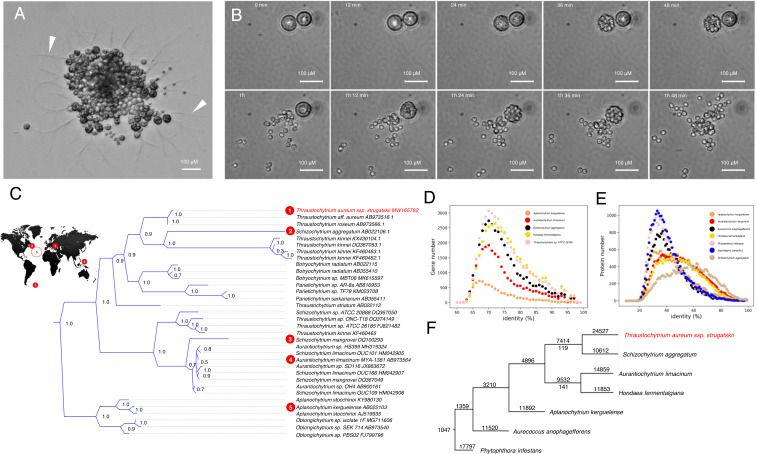
Morphology of *T. aureum ssp. strugatskii*, phylogenetics, and full-genome data comparison. (A) Photomicrograph of the cell aggregate, differential interference contrast. There are cells of different sizes and bothrosome (arrows). (B) Photomicrographs of cell division. Time-lapse photography with 12 min interval, phase contrast. (C) Phylogenetic tree of the closest species based on 18S rRNA sequences. Numbers indicate the bootstrap support for each node. Species with full-transcriptome of full-genome data and their place on tree are marked by red circles: (1) –*T. aureum ssp. strugatskii*, this study, (2-5) BioProject database and JGI (Nordberg et al. 2014). *S. mangrovei* (5) was collected in the area of the Atlantic at an unspecified location. Their place of collection is shown on the map, details in the Supplemental Information 1. (D) Histogram of the BLAST identity distribution between the proteins of the closest species. Bin equals to 1% identity. (E) Histogram of BLAST identity distribution between the genes of the closest species. Bin equals to 1% identity. (F) The phylogenetic tree of *T. aureum ssp. strugatskii* closely related species with transcriptome data. The number of sets of orthologous genes for a particular tree node is indicated on the branches of the tree. The world map and scheme of the sampling sites was based on the Al MacDonald map https://commons.wikimedia.org/wiki/File:World_map_-_low_resolution.svg, available under license https://creativecommons.org/licenses/by-sa/3.0/deed.en.

*T. aureum ssp. strugatskii* morphology is similar to the primary report for *T. aureum* (Goldstein, 1963). They form large cell conglomerates attached to the substrate in a vegetative phase and can form sporangia. We analyzed about 200 cells on each development phase. The diameter of *T. aureum ssp. strugatskii* “cells” is 15–65 µm depending on growing time. Cell aggregates with a distinctive ectoplasmic net formed during prolonged culture for several days (Fig. 1A). After the passage, cells stayed at the growth phase during 30–36 h, consequently forming sporangia with a subsequent transformation into new cells. In some cases, the new daughter cells were immobile and formed cell aggregates later; in other cases, we observed many small motile ellipsoidal zoospores. As a result, each sporangium generated more than 20 proportionally smaller daughter cells. Less than 40 min passed between the first visible morphological changes and complete cell separation (Fig. 1B). Zoospores swam rapidly with frequent changes in direction. The population doubling time was about 4.5 h. The amoeboid cells that are typical for some Thraustochytrids (Bongiorni et al., 2005; Jain et al., 2007; Dellero et al., 2018) were not observed in the developmental cycle of the studied protist.

### Transcriptome assembly

We generated three RNA-seq libraries from the *T. aureum ssp. strugatskii* cell culture, in total 31,679,536 single 150 bp reads (4,751,930,400 nucleotides, GC content 60.3%). We removed low- quality reads and assembled 87.9% reads into 42,366 contigs (maximal length 15,414 bp, N50 1,159 bp). We produced 42,366 contigs from combined libraries, which is 20–39% (Supplemental Information 1). 73% of all reads uniquely mapped to the *T. aureum ssp. strugatskii* transcriptome assembly. We predicted the presence of 85,570 proteins longer than 50 amino acids (Supplemental Information 1).

### Phylogenetic analysis

We performed phylogenetic analysis using the *T. aureum ssp. strugatskii* 18S rRNA sequence. Ninety-eight 18S rRNA genes of 10 family Thraustochytriaceae genera were found using BLAST. The results of the phylogenetic analysis are shown in Fig. 1C and Supplemental Information 2. The closest species are *T. aureum* and *T. roseum* (99.73% sequence similarity with AB022110.1 and AB973566.1, respectively). The partial sequence of the 18S rRNA gene is the only one available for these species. The closest species with genome sequence is *S. aggregatum* (AB022106.1, 84.46% 18S rRNA sequence identity). This species forms an outgroup to *T. kinnei* in the phylogenetic tree. The other representatives of the genus *Schizochytrium* do not form one clade, raising doubts about their belonging to the same genus (Dellero et al., 2018; Ju et al., 2019; Liang et al., 2019). Genus *Thraustochytrium* clusters more compactly but also do not form a separate monophyletic group. Our phylogenetic reconstruction of the Thraustochytriaceae species is in agreement with the previous data (Caamaño et al., 2017), and also points out the necessity of the revision of the family Thraustochytriaceae taxonomy (Dellero et al., 2018; Ju et al., 2019; Liang et al., 2019). Special attention should be given to genera *Schizochytrium* and *Aurantiochytrium* as their representatives are dispersedly distributed around the oceans.

### Comparison of transcriptome and predicted proteome with other species

We compared transcriptome sequences and predicted open reading frames with the ones available in the databases. The distribution of the identity values of *T. aureum ssp. strugatskii* transcriptome sequences and predicted proteome sequences with the closest species are presented in Figs. 1D and 1E and Supplemental Information 3. The closest species by protein sequences is *S. aggregation* with the distribution peak at 62%. It corresponds to the phylogenetic analysis of 18S rRNA sequences. The distribution peak is close to 45% for *H. fermentalgiana* and *A. limacinum*. The amounts of homologous genes between *T. aureum ssp. strugatskii* and KEGG database proteomes are presented in Figs. 1D and 1E. We have found more than 1,700 proteins with identity and coverage of more than 50% with other protists but not with the vertebrates. The phylogenetic tree of species obtained by combining 1047 ortholog trees is shown in Fig. 1F. The number of sets of orthologous genes for a particular tree node is indicated on the branches of the tree. The initial data of the trees and the table of orthologues are attached in the Supplemental Information 2.

### Analysis of proteome and GO terms

For 18,104 proteins of *T. aureum ssp. strugatskii* we showed homology to known organisms. We identified 286 biochemical pathways of protists involving 2,894 proteins from the studied species. Many biochemical pathways contain a complete set of proteins according to KEGG including biosynthesis of fatty acids. There were no protein complexes for vertebrate intercellular contacts, though both *T. aureum ssp. strugatskii* and *H. fermentalgiana* have cadherins (PF00028) which are responsible for calcium-dependent adhesion.

We have found protein domains of enzymes that can decompose organic substrates: amylase (PF00128, PF16657), cellulase (PF00150), lipase (PF01764, PF04083), aspartate protease (PF13650), and xylanase (PF14541, PF14543). These enzymes are also presented in *H. fermentalgiana* (Supplemental Information 4), amylase was previously found only in two (*Aplanochytrium kerguelense* and *S. aggregatum*) out of four species. In addition, *T. aureum ssp. strugatskii* and *H. fermentalgiana* have 290 and 306 WD40 proteins (PF00400), respectively, though the other close species about 200. We revealed a relatively large number of PF14312 and PF17210 proteins binding calcium and phosphatases (PF09423). There were relatively small numbers of leucine-rich repeat proteins (PF12799, PF13516) and kinases (PF01163, PF12330, PF12330, and PF06293) compared to other species (Supplemental Information 4).

**Figure 2:**
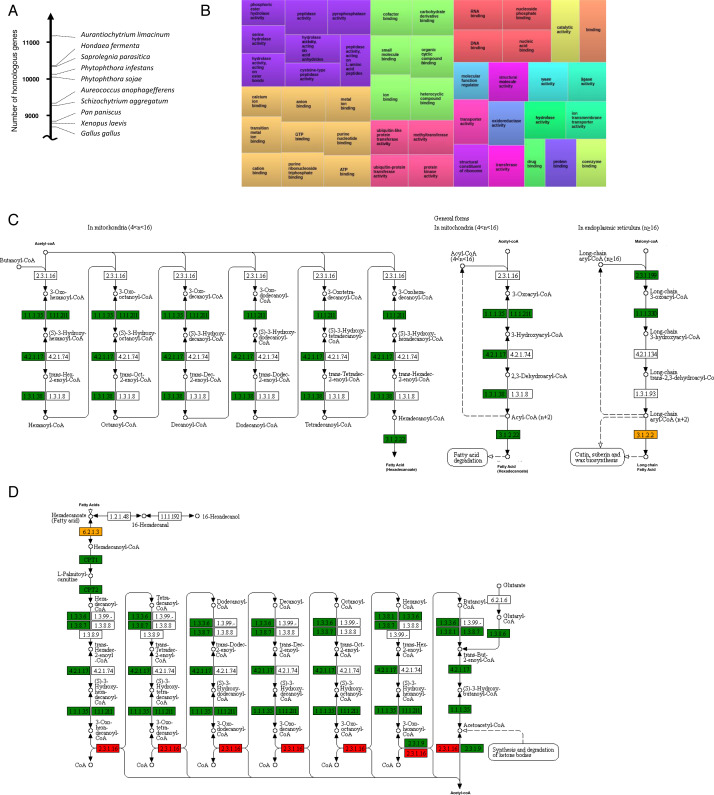
Analysis of the genome and predicted proteome of of *T. aureum ssp. strugatskii*. (A) Homologous proteins between *T. aureum ssp. strugatskii* and the closest species. (B) The frequency of the most represented GO terms. (C) Predicted components of fatty acids synthesis. (D) Predicted components of fatty acids degradation. Visualization of the GO term distribution was made using WEGO 2.0 (http://wego.genomics.org.cn/), visualization of the metabolic pathways is given according to the KEGG database (Kanehisa, 2002).

The protein domain structure was estimated using HMM from Pfam. GO terms were assigned based on protein structure. In total, we revealed 1.699 GO terms (Fig. 2B and Supplemental Information 5). 235 GO terms were observed more than 100 times, such as transition metal ion binding, calcium ion binding, transmembrane transporter activity, oxidoreductase activity, serine-type peptidase activity, and inorganic cation transmembrane transporter activity.

We consider *T. aureum ssp. strugatskii* as a promising source of fatty acids for biotechnology, so in our study, we paid special attention to the fatty acid metabolic pathways. We used sequences of 164 *H. fermentalgiana* proteins (Dellero et al., 2018) and had found 133 homologs in *T. aureum ssp. strugatskii*. These proteins cover all necessary enzymes for fatty acid synthesis and degradation (Figs. 2C–2D, Supplemental Information 4).

## Discussion

A new subspecies of the genus *T. aureum, T. aureum ssp. strugatskii*, reported here, was isolated when attempting to cultivate *in vitro* cell culture of the *B. ovata* from the Black Sea. The species might be associated with *B. ovata*, but this hypothesis needs further studies. *T. aureum ssp. strugatskii* has typical for the family Thraustochytriaceae cell morphology, ability to form cell aggregates, and bothrosome (Moro et al., 2003; Hamamoto & Honda, 2019; Ganuza et al., 2019). We were not able to observe the ameboid form, typical for some Labyrinthulomycetes (Moro et al., 2003; Hamamoto & Honda, 2019; Ganuza et al., 2019).

We used the FAND culture medium based on fetal bovine serum and DMEM. To our knowledge, it is the first usage of a modified mammalian cell culture medium for the culture of protists. This medium is rich in nutrients and more expensive than routinely used ones. Nevertheless, our experience shows that the FAND medium is promising for inclusion in the sets of media for newly discovered protists’ cell cultures.

Labyrinthulomycetes taxonomy is mainly based on molecular phylogenetics. The accepted consensus considering molecular and morphological traits seems to be the separation of Labyrinthulomycetes into two clades: thraustochytrids and labyrinthulids with aplanochytrids (Bennet et al., 2017). Phylogenetic analysis based on 18S rRNA sequence confirms that the reported species belongs to genus *Thraustochytrium* (family Thraustochytriaceae; class Labyrinthulomycetes). We made this conclusion based on good bootstrap support for key nodes. The closest sequences were the ones of *T. aureum* (seven operative taxonomic units) and *T. roseum* (one operative taxonomic unit). All sequences had more than 99% pairwise identity. We were not able to distinguish between *T. aureum* and *T. roseum* by 18S rRNA. Notably, clusterization of the 18S rRNA sequences had not been measured against their species taxonomic position, thus making necessary a previously proposed revision of the family Thraustochytriaceae systematics (Andersen & Ganuza, 2018; Yokoyama & Honda, 2007; Ju et al., 2019; Liang et al., 2019). Our transcriptome data on *Thraustochytrium* close the significant gap in the taxon Thraustochytriaceae. From now on, these data are available for genera *Aplanochytrium*, *Aurantiochytrium*, *Schizochytrium* (two entries), and *Thraustochytrium* (Fig. 1C). In the future, it would allow developing a multilocus genotyping approach for newly described and cultured samples. The used method would be crucial for the revision of the Thraustochytriaceae taxonomy.

Based on proteome analysis we presume that the cadherin (PF00028) protein family could be a possible candidate for calcium-dependent cell adhesion and bothrosome formation. This protein was found in *T. aureum ssp. strugatskii* and *H. fermentalgiana*. The comparison of the proteomes by the previously described method (Wang et al., 2019) revealed the marked proximity of *T. aureum ssp. strugatskii* to *S. aggregation*, but not to *H. fermenta* and *A. limacinum* (Fig. 1B).

Thraustochytrids species are very promising for the production of different fatty acids and their derivatives (Fan et al., 2007; Chang et al., 2012; Abida et al., 2015; Manikan et al., 2015; Aasen et al., 2016; Dellero et al., 2018). For example, squalene (Patel et al., 2019), sterols, and carotenoids (Aki et al., 2003) could be used in the food and cosmetics industries. Also, representatives of the genus *Aurantiochytrium* can double their biomass in 4–5 h and, therefore, are suitable for use in the bioreactors (Min et al., 2012; Taoka et al., 2011) and wastewater treatment (Hipp & Rodríguez, 2018). We identified 133 homologous enzymes that belonged to all fatty acids biosynthesis pathways described in the KEGG database (Fig. 2C). Thus, we consider that *T. aureum ssp. strugatskii* is a promising source of fatty acids for biotechnology, as it is accessible to culture and has a high growth rate. Further experiments are needed to assess its potential for fatty acids production.

Substrate-specificity is one of the important features of the Labyrinthulomycetes ecology (Raghukumar & Damare, 2011; Singh et al., 2014; Pan, Del Campo & Keeling, 2017). Thraustochytrids, as well as Aplanochytrids, have been often found in the plankton samples from various open areas of the Ocean, though it is still a challenge to find a substrate these protists are associated with (Bennet et al., 2017). They may be associated with zooplankton fecal pellets and marine snow (Naganuma et al., 2006; Raghukumar, Ramaiah & Raghukumar, 2001; Damare & Raghukumar, 2008) or may live as passive inhabitants of plankton animal guts or their body surface (Damare & Raghukumar, 2010). Even though jellyfish (Cnidaria & Ctenophora) are widely represented in marine plankton, and their gelatinous consistency may facilitate the attachment or development of Labyrinthulomycetes, no reports of protists living in/on plankton jellyfish have been found in the literature. The fact that we isolated a few species of protists from the *Beroe ovata* animal bodies may indicate such association. Further studies are needed to study the possibility of an association of the Labyrinthulomycetes with pelagic ctenophores or jellyfish. If the protists can actively utilize both waste products and dead remains of gelatinous, this could significantly extend our comprehension of the structure and functioning of the microbial loop as an effective transformer of organic matter in the pelagic ecosystems. Thus, studies of the possible association of Thraustochytrida with gelatinous organisms of the Black Sea seem promising for describing the diversity of this group and assessing its functional role in the ecosystem. To solve this challenge, it is necessary to expand the list of substrates used for studying fungi-like protists in the marine environment to include planktonic jellyfish and ctenophores. The approach for the isolation of Labyrinthulomycetes described in this work could be taken as a basis.

Thus, presented here new protistan subspecies, *T. aureum ssp. strugatskii*, is a promising candidate for the industrial production of organic substances, and therefore essential for the development of biotechnology. Following studies are needed to confirm the association of the protist with pelagic ctenophores, which could be recommended as a new regular substrate in routine studies of micromycetes in the marine environment, in particular, in the Black Sea.

## Conclusions

We isolated and characterized a new subspecies of marine protists, *T. aureum ssp. strugatskii* (family Thraustochytriaceae) from the Black Sea. Transcriptome data for that species and the previously available would facilitate the subsequent multilocus approach of phylogenetic analysis of the family Thraustochytriaceae. The transcriptome analysis revealed that all necessary enzymes for fatty acid synthesis and degradation are present in the studied species. We assume that the protist has a complete gene set for the metabolism of fatty acids and their derivatives. Thus, *T. aureum ssp. strugatskii* could be a promising base for the fast development of bioengineered super producers of these substances for the industry and medicine.

## Supplemental Information

10.7717/peerj.12737/supp-1 Supplemental Information 1 Transcriptome and Proteome sequences of T. aureum ssp. strugatskii

10.7717/peerj.12737/supp-2 Supplemental Information 2 Phylogenetic tree of T. aureum ssp. strugatskii relative species

10.7717/peerj.12737/supp-3 Supplemental Information 3 Available genomes of T. aureum ssp. strugatskii relative species

10.7717/peerj.12737/supp-4 Supplemental Information 4 Pfam domains predicted in T. aureum ssp. strugatskii proteome

10.7717/peerj.12737/supp-5 Supplemental Information 5 GO terms related to T. aureum ssp. strugatskii genes

## Additional Information and Declarations

### Data Availability

The following information was supplied regarding data availability: The data is available at NCBI: MW165782 (18S rRNA); PRJNA669615 (RNA-seq libraries). The FASTA sequences of transcriptome and proteome are also available in the Supplemental File.

### New Species Registration

The following information was supplied regarding the registration of a newly described species: The Thraustochytrium subspecies, Thraustochytrium aureum ssp. strugatskii is registered in MycoBank: 340067. The cell culture was performed at the Collective Center of ICG SB RAS “Collection of Pluripotent Human and Mammalian Cell Cultures for Biological and Biomedical Research” (https://ckp.icgen.ru/cells/; https://www.biores.cytogen.ru/brc_cells/collections/ICG_SB_RAS_CELL). The cell culture is deposited at the Collective Center (THAU1, Cat. No. TAHE00071). The protist is registered in MycoBank: 842785 Name type: Basionym Classification: Chromista Labyrinthulomycota Labyrinthulomycetes Thraustochytriales Thraustochytriaceae Thraustochytrium Epithet: aureum ssp. strugatskii Type specimen or ex type: TAHE00071.
